# Activity Trackers in Physical Therapy for People With Chronic Obstructive Pulmonary Disease in the Netherlands: Cross-Sectional Study on Current Use and Implementation Determinants

**DOI:** 10.2196/59533

**Published:** 2025-02-12

**Authors:** Darcy Ummels, Esther Bols, Roel Johannes Anna Frantzen, Tim Frantzen, Levy Robeerts, Emmylou Beekman

**Affiliations:** 1 Research Centre for Autonomy and Participation of Persons with a Chronic Illness, Academy for Speech and Language Therapy, Zuyd University of Applied Sciences, Heerlen, Netherlands; 2 Department of Rehabilitation Medicine, Care and Public Health Research Institute, Faculty of Health, Medicine & Life Sciences, Maastricht University, Maastricht, Netherlands; 3 Department of Family Medicine, Care and Public Health Research Institute, Faculty of Health, Medicine & Life Sciences, Maastricht University, Maastricht, Netherlands

**Keywords:** physical therapy, activity tracker, implementation, technology, COPD, chronic obstructive pulmonary disease, eHealth, meaningful use, health measurement, active lifestyle, Netherlands, physical activity, barriers, therapy

## Abstract

**Background:**

In the Netherlands, 545,900 people experienced chronic obstructive pulmonary disease (COPD) in 2022. Physical therapy following the Royal Dutch Society for Physiotherapy (Koninklijk Nederlands Genootschap voor Fysiotherapie) guidelines for COPD treatment is a proven effective treatment for people with COPD. The revised guidelines include a new recommendation: a patient’s physical activity level should be assessed with an activity tracker (AT). Literature shows that the implementation of eHealth in clinical practice, in this case, ATs, is challenging.

**Objective:**

This study aims (1) to assess how and why ATs are currently used in physical therapy in patients with COPD and (2) to determine which barriers and facilitators are of relevance for optimal implementation of ATs during the clinical reasoning process of physical therapists in patients with COPD.

**Methods:**

A cross-sectional study was used to evaluate the implementation of ATs in physical therapy. Included participants were physical therapists who were affiliated with Chronisch ZorgNet and had a specialization in COPD treatment. The survey content was based on the Consolidated Framework for Implementation Research, the theory of planned behavior, the framework “experiences of patients with commercially available ATs,” and the Koninklijk Nederlands Genootschap voor Fysiotherapie guidelines for COPD. Physical therapists were questioned via a digital survey.

**Results:**

In total, 211 completed surveys were analyzed. Of the 211 participating physical therapists, 108 (51.2%) used ATs, whereas most of them (n=82, 75.9%) already used ATs before it was advised in the guidelines. Physical therapists indicated that the most important reason to use ATs is that they experience it as an added health care value. Both users and nonusers indicated that the most important reason why they do not use ATs is because their patients do not want to use an AT. The second reason was a lack of knowledge in the nonuser group. Moreover, both users and nonusers indicated that the implementation of ATs was not prepared and planned for within their center.

**Conclusions:**

Overall, these results show that ATs are not yet fully implemented in the Dutch general physical therapy practice in patients with COPD, as recommended by current evidence-based guidelines. Physical therapists need guidance for the successful implementation of ATs. This could be accomplished by providing training for physical therapists, integrating ATs into the education of (future) physical therapists, and providing support during the implementation process of ATs for both the physical therapists and management.

## Introduction

Chronic obstructive pulmonary disease (COPD) is a heterogeneous lung condition characterized by chronic respiratory symptoms (dyspnea, cough, sputum production, and/or exacerbations) due to abnormalities of the airways (bronchitis and bronchiolitis) and/or alveoli (emphysema) that cause persistent, often progressive, airflow obstruction [1,2]. In the Netherlands, 545,900 people experienced COPD in 2022. It is the fourth leading disease in relation to disease burden and the sixth leading disease that causes mortality in the Dutch population [3]. The health care expenses for people with COPD were estimated at US $775 million in 2019, totaling 1% of the total Dutch health care expenses and 23% of the total expenditure for all respiratory diseases [4]. Physical therapy according to the Royal Dutch Society for Physiotherapy (Koninklijk Nederlands Genootschap voor Fysiotherapie [KNGF]) guidelines for COPD treatment [5] is a proven effective treatment for people with COPD. In general, the treatment is a major component of pulmonary rehabilitation and aims to increase a patient’s physical activity level and physical capacity and is accomplished with home exercises and education [5]. The KNGF guidelines describe the clinical reasoning process for physical therapists based on the latest evidence. In the revised guidelines, the new recommendation is that a patient’s physical activity level (eg, number of steps) is measured with an activity tracker (AT). The patient’s physical activity level is an important starting point, as it codetermines whether physical therapy is indicated or a lifestyle coach is sufficient. Therefore, if ATs are not or incorrectly used, the clinical reasoning process is compromised. This could result in misclassification of patients and hence the wrong allocation to physical therapy or lifestyle coaching.

Apart from assigning patients to treatment, ATs can also be used to monitor and evaluate the physical activity level during and after the treatment [6]. Using ATs as an intervention can also enhance the physical activity level of patients with COPD when combined with the guidance of a health care professional [7]. Moreover, they can improve patients’ self-management and self-efficacy [8-10].

Literature shows that the implementation of eHealth in clinical practice, in this case, ATs, is challenging [6,11,12]. Barriers in the implementation of eHealth are among others, the lack of knowledge among health care professionals concerning eHealth possibilities, unclear benefits of eHealth, resistance to change, and clinicians’ perception of impersonal care [13-16]. The degree of actual use of ATs and areas of application by physical therapists in patients with COPD are currently unclear. In addition, more insight into potential barriers and facilitators for successful future implementation of ATs is necessary to optimize the treatment of patients with COPD.

A theoretical framework to assess potential barriers and facilitators for implementing innovations is the Consolidated Framework for Implementation Research (CFIR) [17]. The CFIR consists of 5 domains associated with effective implementation: intervention characteristics, inner setting, characteristics of individuals, outer setting, and process. Besides assessing potential barriers and facilitators for successful implementation, physical therapists need to change their current behavior. The theory of planned behavior (TPB) supports predicting an individual’s intention to engage in a (new) behavior. The TPB consists of 4 aspects: attitude, subjective norms, perceived behavioral control, and intention [18].

This study combines the aspects of the CFIR and the TPB as a background (1) to assess how and why ATs are currently used in physical therapy in patients with COPD and (2) to determine which barriers and facilitators are of relevance for optimal implementation of ATs during the clinical reasoning process of Dutch physical therapists in patients with COPD.

## Methods

A cross-sectional study was performed, in which Dutch physical therapists were questioned via a voluntary digital closed survey.

### Population

Included participants were physical therapists who were affiliated with Chronisch ZorgNet and had a specialization in COPD treatment. Chronisch ZorgNet is a nationwide network that provides training for treating patients with chronic diseases according to the latest evidence (guidelines). The network consists of 3500 physical therapists working in primary, secondary, and tertiary care, of which 1200 physical therapists are specialized in lung physical therapy [19]. This network provided access to this target population. The survey was distributed by emails from Chronisch ZorgNet. All specialized lung physical therapists who were Chronisch ZorgNet members were eligible to participate in this survey. Physical therapists were excluded from this study if they treated less than 1 unique patient with COPD per week.

### Survey

The survey (Multimedia Appendix 1) was drafted from documents based on international scientific literature: (1) the checklist “the use of measurement instruments in clinical practice” [20] based on the CFIR [17] (Q1), (2) the questionnaire “meaningful use of patient-specific goal setting within the clinical reasoning of physical therapists” [21] based on the TPB [18] (Q2), (3) the framework “experiences of patients with commercially available ATs” [22] (Q3), and (4) the KNGF guidelines for COPD [5]. Questions of the checklist Q1 and the questionnaire Q2 were adapted for the application of ATs instead of general measurement instruments. To gain insight into relevant barriers and facilitators, the topics of the framework Q3 were used. The survey was supplemented with questions based on the recommendations for the use of ATs in the COPD guidelines [5]. In total, 9 questions determined the eligibility of the participants (eg, sex, age, and work experience). Based on the question “I use ATs in people with COPD” (yes or no), participants answered either 59 questions if they used ATs or 33 questions if they did not.

Of the 59 questions for the AT users, 26 were based on the checklist “the use of measurement instruments in clinical practice”’ Q1 [20], 5 were based on questionnaire Q2 [21], 20 were based on the framework Q3 [22], and 8 were based on the COPD guidelines [5]. Of the 33 questions for the nonusers, 19 were based on Q1 [20], 5 were based on Q2 [21], and 9 were based on Q3 [22]. The questions were either answered on a 7-point Likert scale (0=totally disagree and 7=totally agree), on a multiple-answer scale, or were open comment questions. Only 4 questions were necessarily answered by yes or no (eg, ATs are provided to our patients: yes or no). Participants were able to review and change their answers before completing the survey.

To ensure the content validity of the combined questionnaires, the draft survey was sent to 5 experts for feedback on the usability and face validity of the survey: 2 experts in the field of measurement tools in health care, 1 expert in the field of ATs in health care, and 2 specialized physical therapists. The feedback required no changes to the survey and the final survey was constructed (Multimedia Appendix 1).

Data collection was performed in May 2021. The survey was completed via clickable link, and QR code in the email leading to the survey in QuestBack Essentials (2021). For each question, a digital page emerged; in total, there were between 33 and 59 pages excluding the start- and the end page and 7 questions regarding population characteristics. All questions were mandatory questions and needed to be completed before the survey automatically continued. One reminder was sent after 1 week. Participants were asked to answer the questions as accurate as possible, and it was explicitly addressed that the results were analyzed anonymously. Data were stored in a secured data repository of Zuyd University of Applied Sciences for 15 years, and only the participating researchers had access to these data.

### Data Analyses

Data analyses were performed using SPSS Statistics (version 23.0; IBM Crop). Only fully completed surveys were analyzed. Descriptive statistics of the participants’ characteristics were presented as a number (percentage) for the categorical variables sex, workplace setting, and whether they completed a recent COPD course. For the continuous variables, age, work experiences, and the number of treated unique patients with COPD per week were presented as a mean (SD). Answers to questions with a 7-point Likert scale were presented as mean (SD). Responses to open comment questions were treated as qualitative data and categorized into themes by using directed content analyses via inductive coding [23] and presented as a number (percentage) per theme. Open answers that did not fit any code were labeled as other. Responses were separately analyzed for users and nonusers of ATs. In case of any missing data, listwise deletion was applied.

### Ethical Considerations

No ethics approval was required since the research was not subject to the Medical Research Involving Human Subjects Act (Wet medisch-wetenschappelijk onderzoek met mensen). The research was part of continuous quality monitoring and improvement of daily COPD care within a national quality network (Chronisch ZorgNet). This study did not require written informed consent. Participating physical therapists were informed about the purpose of this survey prior to commencing the survey. Physical therapists voluntarily participated in this study. Study results were processed anonymously. Physical therapists were always able to end the survey without any consequences. There was no compensation for the physical therapists. The CHERRIES (Checklist for Reporting Results of Internet E-Surveys) was used (Checklist 1).

## Results

### Population Characteristics

In total, 216 physical therapists completed the survey. Five physical therapists were excluded because they did not meet the inclusion criteria (treating less than 1 unique patient with COPD per week), leaving 211 responses for analysis. There were no missing data. The characteristics of the physical therapists are displayed in Table 1.

**Table 1.** Characteristics of the participating physical therapist (N=211).

Characteristic	Participants
Sex (male), n (%)	74 (35.1)
Age (years), mean (SD)	46.5 (11.7)
Work experience (years), mean (SD)	22.8 (11.1)
Work setting, n (%)	
Primary care	197 (93.5)
Secondary care	5 (2.5)
Primary and secondary care	8 (3.8)
Tertiary care	1 (0.5)
Number of unique patients with COPD^a^ per week, mean (SD)	8.2 (6.5)
Completed a recent COPD course, n (%)	208 (98)

a COPD: chronic obstructive pulmonary disease.

Results of the survey are presented per research questions.

### How ATs Are Used

Of the 211 participating physical therapists, 108 (51.2%) used an AT, whereas most of them (n=82, 75.9%) already used an AT before it was advised in the guidelines. Most physical therapists measured the number of steps (n=64, 55.2%) and used a standard app on a smartphone (n=97, 89.8%). Other parameters named were, for example, activities of daily living, distance, physical capacity, and heart rate. The 2 most common goals for the use of an AT were to inventory the physical activity level (n=94, 87%) and to stimulate the physical activity level of the patient (n=88, 81.5%). All results on how ATs were used in physical therapy in patients with COPD are presented in Table 2.

**Table 2.** Results on how activity trackers (ATs) are currently used in physical therapy in patients with chronic obstructive pulmonary disease (COPD; N=211; based on digital survey).

Question	User (n=108)	Nonuser (n=103)
I use AT in people with COPD, n (%)	Yes: 108 (100)^a^	No: 103 (100)^a^
Before the release of the COPD guidelines, I already used AT in people with COPD, n (%)	Yes: 82 (75.9) No: 26 (24.1)	—^b^
It is clear what I want to measure with an AT^c^, mean (SD)	5.8 (1.3)	—
Which parameter or concept do you measure with an AT? n (%)	Number of steps: 64 (55.2) Physical activity: 43 (37.1) Other^d^: 9 (7.7)	—
It is clear to me with which AT I can measure these parameters^c^, mean (SD)	5.3 (1.5)	—
There are agreements within our center about the choice of the used type of AT^c^, mean (SD)	3.2 (1.9)	—
Within our center, we use the following type of AT, n (%)	App on a smartphone: 97 (89.8) Commercially available pedometer: 57 (52.7) Commercially available accelerometer: 14 (12.9) Already owned by patient: 2 (1.8)	—
Within our center, we use the following brand AT, n (%)	Standard app on a smartphone: 26 (24.1) Unknown brand: 23 (21.3) Already owned by patient: 14 (12.9) Fitbit: 12 (11.1) Omron: 8 (7.4) Other^d^: 7 (6.4) Yamax: 3 (2.7) McRobberts: 2 (1.8)	—
The AT is worn by people with COPD on the following locations, n (%)	Trouser pocket: 88 (81.4) Wrist: 52 (48.1) Hip: 13 (12) Bag: 13 (12) Other^d^: 5 (4.6) Chest pocket: 4 (3.7) Lower back: 2 (1.8) Ankle: 2 (1.8)	—
Which brand of AT would you recommend to your patients? n (%)	Do not know: 49 (45.3) Apps: 23 (21.2) Fitbit: 21 (19.2) I do not recommend a brand: 6 (5.5) Garmin: 5 (4.6) Other: 5 (4.6) Apple: 4 (3.7)	—
I stimulate my patients to purchase an AT^c^, mean (SD)	4.9 (1.4)	—
At this moment, in how many unique patients with COPD per week are you using an AT? mean (SD)	5.5 (4.2)	—
I use AT for the following goals in patients with COPD, n (%)	Inventory: 94 (87) Stimulation: 88 (81.5) Allocating: 64 (59.2) Evaluation: 60 (55.5)	—
My patients with COPD wear an AT for the recommended number of days (7 days)^c^, mean (SD)	4.4 (1.5)	—

a Of the 211 respondents, 108 (51.2%) answered “yes” and 103 (38.8) answered “no.”

b Not available.

c Likert scale with a range of 0‐7 (0=totally disagree and 7=totally agree).

d Multiple open answers which were categorized as other.

### Why ATs Are Used

Physical therapists who used ATs because they believed that it is an added value to health care (n=46, 42.5%) and because the guidelines recommend to use ATs (n=44, 40.7%). Physical therapists considered the cutoff value of 5000 steps per day for patients with COPD to be a realistic measure of sufficient activity (mean 4.7, SD 1.5). The primary reason physical therapists did not use ATs both among users (n=58, 53.7%) and nonusers (n=35, 33.9%) was that patients themselves indicated that they did not want to use them. In the nonusers group, lack of knowledge (n=31, 30.9%) was the second important reason for nonuse. Other reasons mentioned were, for example, bad experiences, difficulty implementing ATs, and concerns about the clinometric properties of the ATs.

In most centers, there was no consensus on why they were going to use ATs (mean 3.4, SD 1.8). Users scored high on questions regarding whether ATs contributed to the assessment of physical activity levels (mean 5.8, SD 1.1), allocation of patients into profiles (mean 5.8, SD 1.1), evaluation of physical activity levels (mean 5.9, SD 0.9), and stimulation of physical activity levels (mean 5.9, SD 1.0). Results on why ATs were used in physical therapy in patients with COPD are presented in Table 3.

**Table 3.** Results on why activity trackers (ATs) are currently used in physical therapy in patients with chronic obstructive pulmonary disease (COPD; N=211; based on digital survey).

Question	User (n=108)	Nonuser (n=103)
Within our center, we have agreed on why we (are going to) use AT^a^, mean (SD)	3.4 (2.0)	3.2 (1.9)
The choice for the concerned AT was made based on, n (%)	Availability: 73 (67.6) Costs: 49 (45.3) Feasibility: 43 (39.8) Own experience: 19 (17.5) Validity: 15 (13.8) Already owned by the center: 5 (4.6) Other^b^: 6 (5.5%)	—^c^
I want to measure the following specific activities, n (%)	Walking: 105 (97.2) Bicycling: 52 (48.1) Running: 11 (10.1) Activities of daily living: 8 (7.4) Swimming: 7 (6.4) Other^b^: 5 (4.6) Walking stairs: 2 (1.8)	—
I want to measure the following parameters, n (%)	Number of steps: 104 (96.2) Active minutes: 73 (67.5) Walked distance: 53 (49) Heart rate: 35 (32.4) Passive minutes: 34 (31.4) Calories: 13 (11.1) Other^b^: 3 (2.7)	—
I think the cutoff value of 5000 steps per day to be sufficiently active is a realistic cutoff value^a^, mean (SD)	4.7 (1.5)	—
The most important reason I am using an AT in patients with COPD is, n (%)	Because it is an added health care value: 46 (42.5) Because of the guidelines: 44 (40.7) Own interest: 9 (8.3) Other^b^: 5 (4.6) Because it is mandatory in my network: 4 (3.7)	—
When I use an AT, it contributes to the inventory of the physical activity level and the physiotherapeutic diagnosis of a patient^a^, mean (SD)	5.8 (1.1)	—
When I use an AT, it contributes to allocating patients into the profiles of the guidelines^a^, mean (SD)	5.8 (1.1)	—
When I use an AT, it contributes to evaluating the physical activity during the treatment process in patients with COPD^a^, mean (SD)	5.9 (0.9)	—
When I use an AT, it contributes to stimulating physical activity as part of my intervention^a^, mean (SD)	5.9 (1.0)	—
I think AT should be used in patients with COPD^a^, mean (SD)	5.2 (1.7)	4.3 (1.7)
My patients with COPD think it is important to (start) using AT^a^, mean (SD)	4.1 (1.2)	3.9 (2.0)
My patients with COPD are more aware of their functioning by using an AT^a^, mean (SD)	5.7 (1.1)	5.8 (1.4)
Health care centers, comparable to my center are using AT^a^, mean (SD)	4.2 (0.9)	3.3 (1.2)
External organizations are obligating the use of AT^a^, mean (SD)	3.4 (1.8)	2.6 (1.6)
The most important reason I do not use AT in some patients with COPD is, n (%)	Patients do not want to: 58 (53.7) No added value: 13 (12) No knowledge: 9 (8.3) No AT available: 8 (7.4) Costs: 7 (6.4) Other^b^: 6 (5.5)	Patients do not want to: 35 (33.9) No knowledge: 31 (30.9) No AT available: 9 (8.7) Bad experience: 8 (7.7) Other^b^: 8 (7.7) No added value: 7 (6.7) Costs: 7 (6.7)

a Likert scale with a range of 0‐7 (0=totally disagree and 7=totally agree).

b Multiple open answers which were categorized as other.

c Not available.

### Barriers and Facilitators for Implementation of ATs

No remarkable differences exist between users and nonusers regarding the experienced barriers and facilitators. For both groups, a relevant barrier to using ATs was the costs (users: n=75, 69.4% vs nonusers: n=77, 74.7%) and the cognitive or communication skills of the patient (users: n=75, 69.4% vs nonusers: n=50, 48.5%). Other reasons that were mentioned were prioritizing the patient over scientific pursuits, privacy concerns, and AT fails to measure parameters that therapists find meaningful. Important facilitators in both groups for using ATs were the motivation of the patient (users: n=90, 83.3% vs nonusers: n=70, 67.9%) and the user-friendliness of the AT (users: n=64, 59.2% vs nonusers: n=57, 55.3%). Both users (mean 3.6, SD 1.8) and nonusers (mean 2.5, SD 1.6) indicated that they lack the knowledge to provide a recommendation of commercially available ATs to their patients. Likewise, both groups indicated insufficient education or coaching possibilities for the health care professionals on ATs within their center (users: mean 3.7, SD 1.9 and nonusers: mean 2.7, SD 1.8). Further barriers and facilitators are presented in Table 4.

**Table 4.** Results on relevant barriers and facilitators for optimal implementation of activity trackers (ATs) during the clinical reasoning process of Dutch physical therapists in patients with chronic obstructive pulmonary disease (COPD; N=211; based on digital survey).

Question	User (n=108)	Nonuser (n=103)
I have sufficient knowledge to recommend commercially available AT to my patients regarding price, functions, and quality^a^, mean (SD)	3.6 (1.8)	2.5 (1.6)
I am capable to draft personalized physical activity goals together with my patient^a^, mean (SD)	5.8 (1.0)	5.8 (1.3)
I am capable to teach my patients about the functions, use, and interpretations of AT^a^, mean (SD)	5.4 (1.3)	4.9 (1.8)
ATs are provided to our patients, n (%)	No: 71 (65.7) Yes: 37 (34.3)	No: 99 (96.1) Yes: 4 (3.9)
The following factors stimulate the use of AT during the clinical reasoning in patients with COPD, n (%)	Motivation for the patient: 90 (83.3) User-friendliness: 64 (59.2) The added value: 59 (54.6) Reliability and validity: 38 (35.1) Costs: 28 (25.9) Time investment: 25 (23.1)	Motivation for the patient: 70 (67.9) User-friendliness: 57 (55.3) The added value: 54 (52.4) Reliability and validity: 40 (38.8) Costs: 25 (24.2) Time investment: 18 (17.4) Other^b^: 5 (4.8)
The following factors obstruct the use of AT during clinical reasoning in patients with COPD, n (%)	Costs: 75 (69.4) Cognitive or communication skills of the patient: 75 (69.4) Time investment for the therapist: 37 (34.2) User-friendliness: 36 (33.3) Time investment for the patient: 34 (31.4) Reliability and validity: 26 (24) Other^b^: 3 (2.7)	Costs: 77 (74.7) Cognitive or communication skills of the patient: 50 (48.5) Time investment for the therapist: 46 (44.6) User-friendliness: 45 (43.6) Reliability and validity: 40 (38.8) Time investment for the patient: 40 (38.8) Other^b^: 7 (6.7)
I know how I can measure physical activity with an AT^a^, mean (SD)	5.5 (1.4)	—^c^
I know how I can interpret the results of the AT^a^, mean (SD)	5.4 (1.2)	—
I know how I can discuss the results of the AT with my patients^a^, mean (SD)	5.8 (1.0)	—
The use of AT is part of our mission and vision and is incorporated into our policy^a^, mean (SD)	5.2 (2.0)	3.8 (2.3)
The use of AT fits in our care process^a^, mean (SD)	5.6 (1.2)	4.6 (1.9)
There is sufficient support and involvement from the management when using AT^a^, mean (SD)	5.2 (1.9)	4.1 (1.9)
In our center, there is someone who guides the implementation process of AT^a^, mean (SD)	2.7 (2.0)	2.2 (1.7)
In our center, there is sufficient education or coaching for the health care professionals about AT^a^, mean (SD)	3.7 (1.9)	2.7 (1.8)
In our center, there is sufficient time to try to use AT^a^, mean (SD)	4.0 (1.9)	3.4 (1.7)
In our center, the use of AT is incorporated in our electronic patients’ files^a^, mean (SD)	4.5 (2.2)	3.4 (2.3)
In our center, there are sufficient AT available, n (%)	No: 63 (58.3) Yes: 24 (22.2) Not applicable: 20 (18.5)	No: 67 (65) Yes: 5 (4.8) Not applicable: 21 (20.3)
I am open to (start to) using AT in patients with COPD^a^, mean (SD)	6.5 (0.8)	5.8 (1.4)
I am planning to continue or start using AT in patients with COPD^a^, mean (SD)	6.4 (0.8)	5.2 (1.5)
I have sufficient knowledge to (start to) use AT^a^, mean (SD)	5.5 (1.3)	4.3 (1.7)
I trust I can use AT in patients with COPD^a^, mean (SD)	5.5 (1.2)	4.8 (1.7)
My colleagues are open to (start) using an AT^a^, mean (SD)	5.5 (1.5)	5.2 (2.0)
My colleagues have sufficient skills and knowledge to (start) using AT^a^, mean (SD)	5.1 (1.7)	4.7 (2.2)
My patients with COPD are open to (start) using AT^a^, mean (SD)	4.6 (1.1)	4.0 (1.8)
My patients with COPD have sufficient skills and knowledge to (start) using an AT^a^, mean (SD)	4.0 (1.4)	3.6 (2.0)
My patients with COPD experience commercially available AT as technical complex^a^, mean (SD)	5.1 (1.3)	5.7 (1.8)
In our center, the implementation of AT was prepared and planned^a^, mean (SD)	3.5 (2.3)	2.7 (2.4)
In our center, we started using AT on a trial basis and started to adapt our care process^a^, mean (SD)	4.2 (1.9)	2.2 (1.5)
In our center, relevant persons were involved in the implementation process: health care professionals, patients (representatives), and supporting professionals (policy)^a^, mean (SD)	3.4 (2.0)	2.9 (2.5)
In our center, the use of AT are frequently evaluated and improved^a^, mean (SD)	3.0 (1.9)	2.9 (2.8)

a Likert scale with a range of 0‐7 (0=totally disagree and 7=totally agree).

b Multiple open answers which were categorized as other.

c Not available.

## Discussion

### Principal Findings

This research aimed to assess how and why ATs are currently used in physical therapy in patients with COPD and to determine which barriers and facilitators are of relevance for optimal implementation of ATs during the clinical reasoning process of Dutch physical therapists.

It is known that the implementation of an innovation is often challenging [11]. However, of the 211 participating physical therapists, 108 (51.2%) used an AT, and most of them (n=82, 75.9%) already used an AT before it was advised in the guidelines. This relatively high percentage could be explained by the fact that physical therapists indicated that the most important reason for using ATs is the experienced added health care value. Another explanation could be that the included physical therapists were affiliated with Chronisch ZorgNet and might therefore be more sensitized to provide evidence-based care. Furthermore, it was notable that regardless whether a physical therapist was a user or nonuser, the barriers and facilitators for using ATs were roughly the same. Both users and nonusers indicated that the most important reason for not using ATs is because their patients do not want to use an AT. However, it is debatable whether this is an assumption of the physical therapist or the actual opinion of the patient, as a recent review describes that patients with COPD in general enjoyed using ATs and found using the ATs easy [24]. The second (nonusers) and third (users) reason for physical therapists to not use ATs was a general lack of knowledge. Physical therapists indicated that they did not know which brand of commercially available AT they could recommend to their patients, which is related to the experienced lack of knowledge regarding price, functions, and quality. In contradiction, knowledge regarding how to measure with ATs, how to interpret the data of the AT, and how to discuss the data of the AT with a patient was scored high by the physical therapists. A possible explanation could be that therapists lack different knowledge: the knowledge on how to measure with ATs, how to interpret the data of the AT, and how to discuss the data of the AT.

### Comparison to Prior Work

The general adaption rate of eHealth in physical therapy seems to be similar to the adaption rate of 52% found in this study [25,26]. However, the use of the ATs in this study was self-reported; therefore, the presence of biases in the reported use and perception of ATs might be present. Most physical therapists used an app on a smartphone as an AT, which links to the fact that availability and costs were important factors when selecting an AT. Most smartphones automatically measure step count, and apps are mostly free of use. However, the validity of apps regarding measuring physical activity is low, especially in older adults during low walking speed, as is the case in activities of daily living [27]. Most physical therapists considered the cutoff value of 5000 steps per day to be sufficiently active, used in the new national guidelines, to be realistic. This is valuable information, considering that this cutoff value in the revised guidelines is based on previous studies. Several studies mention a minimum of 7500‐10,000 steps per day in order to have a healthy active lifestyle [28-30]. However, it seems that a healthy person reaches only 5500‐6000 steps per day on average, and people with a chronic disease 3500‐5500 steps per day [29-31]. Consequently, in the national guidelines, a cutoff value of 5000 steps per day is used for people with COPD to be sufficiently active [32,33]. This study suggests that 5000 steps per day might be a minimal clinical important threshold in daily physical therapy practice.

Both users and nonusers indicated that the implementation of ATs was not prepared and planned for within their center. To successfully implement eHealth, internal and external facilitation, audit and feedback, management support, and training of clinicians are essential according to a recent systematic review [34] and are in line with the CFIR [17]. Wilde et al [35] similarly showed the physical therapists’ need for guidance and information to support the integration of ATs within the practice. A solution could be to provide education and training to management and physical therapists. Management should be trained to guide an implementation trajectory; however, the use of implementation support practitioners could also be an option [36]. Implementation support practitioners are professionals who support organizations, leaders, and staff in their implementation of evidence-informed practices and policies [37]. Current physical therapists and physical therapy students should be educated and trained. To optimize the adoption of ATs in clinical settings, educational interventions should be developed based on the best available evidence, clinical expertise, and patient values and circumstances [38].

However, it is important to train physical therapists’ and students’ general eHealth competencies in order to use eHealth in a broader spectrum than ATs only. It is suggested that education consists of 5 competency themes: information communication technology attitudes and skills, interpretation and analysis of eHealth data, support and guidance, communication skills, and privacy and confidentiality [39]. Current health care professionals are insufficiently trained in all of these competencies and therefore struggle to perform these skills in their daily clinical practice [40]. A better understanding and more digital competency could also lead to better experiences and better outcomes. On the other hand, a previous study showed that patients with chronic diseases (including COPD) experience ATs as technically complicated and wish to receive support by their physical therapist during the use within a diagnostic and/or therapeutic process [22]. Currently, several (digital) tools and websites are available to support health care professionals in the goal-oriented selection of eHealth [41,42].

### Strengths and Limitations

Several limitations of this study need to be mentioned. First, this study addressed technology acceptance on a limited basis. The level of technology acceptance might influence whether ATs are used or not. Studies showed that if health care professionals are already experienced eHealth users, they report fewer implementation barriers and experience more advantages (eg, a more positive attitude toward eHealth) [43]. Second, participating physical therapists were affiliated with Chronisch ZorgNet, an organization in which physical therapists treating patients with chronic diseases are trained to work evidence-based. This might have biased the results since affiliated physical therapists might be more prone to adhere to recent guidelines and be motivated to implement innovations. On the other hand, all participating physical therapists were members of Chronisch ZorgNet, which includes almost all physical therapists who are specialized in lung physical therapy. Therefore, the study sample constitutes a representative sample for primary care. A strength of this study is that the survey was based on relevant international scientific literature and models in relation to implementation and behavioral change (CFIR and TPB), which enables generalization beyond the Dutch context [5,13-18,22,44].

### Conclusions

Overall, these results show that ATs are not yet fully implemented in the Dutch general physical therapy practice in patients with COPD, as recommended by current evidence-based guidelines. Both users and nonusers indicated that the most important reason for not using ATs is because their patients do not want to or at least the health care professionals experienced it that way. However, it is debatable whether this is an assumption of the physical therapist or the actual opinion of the patient. Physical therapists need guidance for the successful implementation of ATs. This could be accomplished by providing training for current physical therapists, integrating ATs into the education of (future) physical therapists, and providing support during the implementation process of ATs for both the physical therapists and management.

## Supplementary material

10.2196/59533 Multimedia Appendix 1 Questionnaire for both users and nonusers.

10.2196/59533 Checklist 1 CHERRIES (Checklist for Reporting Results of Internet E-Surveys).

## Abbreviations

AT: activity tracker
CFIR: Consolidated Framework for Implementation Research
CHERRIES: Checklist for Reporting Results of Internet E-Surveys
COPD: chronic obstructive pulmonary disease
KNGF: Koninklijk Nederlands Genootschap voor Fysiotherapie
TPB: theory of planned behavior
