# Supplementary material for: Activity Trackers in Physical Therapy for People With Chronic Obstructive Pulmonary Disease in the Netherlands: Cross-Sectional Study on Current Use and Implementation Determinants
Source: JMIR Form Res. 2025 Feb 12;9:e59533. doi: 10.2196/59533 (PMC11838813; doi:10.2196/59533)
Supplement: Multimedia Appendix 1 [file formative-v9-e59533-s001.docx]

| **Item** | **Question** | **Answer option** | **Based on literature** |
| --- | --- | --- | --- |
| 1 | I use activity trackers in people with COPD. | - Yes 🡪 questions 2-60 - No 🡪 questions 60-102 | 1,2 |
| 2 | Before the release of the COPD guideline of the Royal Dutch Society for Physiotherapy, I already used activity trackers in people with COPD. | - Yes - No | 1,2,6,7 |
| 3 | It is clear what (parameter and concept) I want to measure with an activity tracker. | 7-point Likert scale (Strongly disagree- Strongly agree) | 1,2 |
| 4 | Which parameter or concept do you measure with an activity tracker? | Open | 1,2 |
| 5 | It is clear to me with which activity tracker I can measure these parameters. | 7-point Likert scale (Strongly disagree- Strongly agree) | 1,2 |
| 6 | There are agreements within our centre about the choice of the used type of activity tracker. | 7-point Likert scale (Strongly disagree- Strongly agree) | 1,2 |
| 7 | Within our centre, we use the following type of activity tracker. | - Commercially available app on a smartphone - Commercially available pedometer - Commercially available accelerometer - Research-grade accelerometer - Other: | 5 |
| 8 | Within our centre, we use the following brand activity tracker: | Open | 5 |
| 9 | The choice for the concerned activity tracker was made based on: | Multiple-choice:   - Costs - Validity - Feasibility - Availability for patient - Own experience - Other: | 5 |
| 10 | The activity trackers are worn by people with COPD on the following locations: | - Trouser pocket - Wrist - Hip - Upper arm - Bag - Other: | 5 |
| 11 | Which brand of activity tracker would you recommend to your patients? | Open | 5 |
| 12 | I stimulate my patients to purchase an activity tracker. | 7-point Likert scale (Strongly disagree- Strongly agree) | 5 |
| 13 | In our centre we agreed to use activity trackers for the following goals: | Multiple-choice:   - To categorize the patients into the guideline’s profiles - Inventory tool for physical activity - Evaluation tool for physical activity - Intervention tool to stimulate physical activity - Other: | 5 |
| 14 | I know how I can measure physical activity with an activity tracker. | 7-point Likert scale (Strongly disagree- Strongly agree) | 1,2,8-10 |
| 15 | I want to measure the following specific activities: | - Swimming - Bicycling - Walking - Running - Other: | 5 |
| 16 | I want to measure the following parameters: | Multiple-choice:   - Number of steps - Active minutes - Passive minutes - Calories - Walked distance - Heartrate - Other: | 5 |
| 17 | I know how I can interpret the results of the activity tracker. | 7-point Likert scale (Strongly disagree- Strongly agree) | 1,2,8-10 |
| 18 | I think the cut-off value of 5000 steps per day to be sufficiently active is a realistic cut-off value. | 7-point Likert scale (Strongly disagree- Strongly agree) | 5,6,7 |
| 19 | I know how I can discuss the results of the activity tracker with my patients. | 7-point Likert scale (Strongly disagree- Strongly agree) | 1,2,8,9 |
| 20 | At this moment, in how many unique patients are you using an activity tracker? | Open | 5 |
| 21 | The most important reason I am using an activity tracker in COPD patients is: | - Because the COPD guideline of the Royal Dutch Society for Physiotherapy recommends it - Because of my interest - Because my patients want to use an activity tracker - Because it is an added value in the treatment of the patient - Because it is mandatory in my association - Because we agreed to use activity trackers in my centre - Other: | 5,11 |
| 22 | I use activity trackers for the following goals in patients with COPD: | - Inventory of the physical activity of the patient - To categorize patients according to the guideline’s categories. - Evaluating the treatment goals regarding physical activity - Stimulating physical activity in patients with COPD - Other: | 6,7 |
| 23 | The most important reason I don’t use activity trackers in some COPD patients is: | - I have no knowledge about activity trackers - Bad experience with activity trackers - No added value in the treatment - Patients don’t want to use an activity tracker - Other: | 3,4,8-11 |
| 24 | I am open to (start to) using activity trackers in COPD patients. | 7-point Likert scale (Strongly disagree- Strongly agree) | 1,2,8-10 |
| 25 | I am planning to keep/start using activity trackers in COPD patients. | 7-point Likert scale (Strongly disagree- Strongly agree) | 3,4 |
| 26 | I think activity trackers should be used in COPD patients. | 7-point Likert scale (Strongly disagree- Strongly agree) | 3,4 |
| 27 | I have sufficient knowledge to (start to) use activity trackers. | 7-point Likert scale (Strongly disagree- Strongly agree) | 1,2,8-10 |
| 28 | I have sufficient knowledge to recommend commercially available activity trackers to my patients regarding price, functions and quality. | 7-point Likert scale (Strongly disagree- Strongly agree) | 5,8-10 |
| 29 | I trust I can use activity trackers in COPD patients. | 7-point Likert scale (Strongly disagree- Strongly agree) | 3,4,8-10 |
| 30 | I am capable to draft personalized physical activity goals together with my patient. | 7-point Likert scale (Strongly disagree- Strongly agree) | 5 |
| 31 | I am capable to teach my patients about the functions, use and interpretations of activity trackers. | 7-point Likert scale (Strongly disagree- Strongly agree) | 5,8-10 |
| 32 | My colleagues are open to (start) using an activity tracker. | 7-point Likert scale (Strongly disagree- Strongly agree) | 1,2,10 |
| 33 | My colleagues have sufficient skills and knowledge to (start) using activity trackers. | 7-point Likert scale (Strongly disagree- Strongly agree) | 1,2 |
| 34 | My COPD patients are open to (start) using activity trackers. | 7-point Likert scale (Strongly disagree- Strongly agree) | 1,2,10 |
| 35 | My COPD patients think it is important to (start) using activity trackers. | 7-point Likert scale (Strongly disagree- Strongly agree) | 5,10 |
| 36 | My COPD patients wear an activity tracker the recommended number of days (7 days). | 7-point Likert scale (Strongly disagree- Strongly agree) | 6,7 |
| 37 | My COPD patients have sufficient skills and knowledge to (start) using an activity tracker. | 7-point Likert scale (Strongly disagree- Strongly agree) | 1,2,10 |
| 38 | My COPD patients experience commercially available activity trackers as technical complex. | 7-point Likert scale (Strongly disagree- Strongly agree) | 5,10 |
| 39 | My COPD patients are more aware of their functioning by using an activity tracker. | 7-point Likert scale (Strongly disagree- Strongly agree) | 3,4 |
| 40 | When I use an activity tracker it contributes to the inventory of the physical activity level and the physiotherapeutic diagnosis of a patient. | 7-point Likert scale (Strongly disagree- Strongly agree) | 5,11 |
| 41 | When I use an activity tracker it contributes to categorizing the patients into the profiles of the guideline. | 7-point Likert scale (Strongly disagree- Strongly agree) | 5,11 |
| 42 | When I use an activity tracker it contributes to evaluating the physical activity during the treatment process in patients with COPD. | 7-point Likert scale (Strongly disagree- Strongly agree) | 5,11 |
| 43 | When I use an activity tracker it contributes to stimulating the physical activity as part of my intervention. | 7-point Likert scale (Strongly disagree- Strongly agree) | 5,11 |
| 44 | The use of activity trackers is part of our mission and vision and is incorporated into our policy. | 7-point Likert scale (Strongly disagree- Strongly agree) | 1,2,8,9 |
| 45 | The use of activity trackers fits in our care process. | 7-point Likert scale (Strongly disagree- Strongly agree) | 1,2,8,9,11 |
| 46 | There is sufficient support and involvement from the management when using activity trackers. | 7-point Likert scale (Strongly disagree- Strongly agree) | 1,2,8,9 |
| 47 | In our centre, there is someone who guides the implementation process of activity trackers. | 7-point Likert scale (Strongly disagree- Strongly agree) | 1,2,9 |
| 48 | In our centre, there is sufficient education/coaching for the healthcare professionals about activity trackers. | 7-point Likert scale (Strongly disagree- Strongly agree) | 1,2,8,9 |
| 49 | In our centre, there is sufficient time to try to use activity trackers. | 7-point Likert scale (Strongly disagree- Strongly agree) | 1,2,8,9 |
| 50 | In our centre, the use of activity trackers is incorporated in our electronic patients' files. | 7-point Likert scale (Strongly disagree- Strongly agree) | 1,2,8,9 |
| 51 | Activity trackers are provided to our patients. | - No - Yes; for how many days? | 5,8,9,11 |
| 52 | In our centre, there are sufficient activity trackers. | - No - Yes | 5,8,9,11 |
| 53 | To my centre, comparable healthcare centres are using activity trackers. | 7-point Likert scale (Strongly disagree- Strongly agree) | 1,2,9 |
| 54 | External organizations are obligating the use of activity trackers. | 7-point Likert scale (Strongly disagree- Strongly agree) | 1,2,9 |
| 55 | In our centre, the implementation of activity trackers was prepared and planned. | 7-point Likert scale (Strongly disagree- Strongly agree) | 1,2,9 |
| 56 | In our centre, we started using activity trackers on a trial basis and started to adapted our care process. | 7-point Likert scale (Strongly disagree- Strongly agree) | 1,2,9 |
| 57 | In our centre relevant persons were involved in the implementation process: healthcare professionals, patients (representatives), supporting professionals (policy). | 7-point Likert scale (Strongly disagree- Strongly agree) | 1,2,8,9 |
| 58 | In our centre, the use of activity tracker are frequently evaluated and improved. | 7-point Likert scale (Strongly disagree- Strongly agree) | 1,2 |
| 59 | The following factors stimulate the use of activity trackers during the clinical reasoning in patients with COPD: | Multiple-choice:   - Costs - Time investment for the therapist - Reliability and validity - User-friendliness - Motivation for the patient - The added value for the clinical reasoning - Other: | 5,8-11 |
| 60 | The following factors obstruct the use of activity trackers during the clinical reasoning in patients with COPD: | Multiple-choice:   - Costs - Time investment for the therapist - Reliability and validity - User-friendliness - Time investment for the patient - Cognitive/ communication skills of the patient - Other: | 5,8-11 |
| 61 | The most important reason I am not using activity trackers is: | - I have no knowledge about the use of activity trackers - Bad experiences with activity trackers - No added value to the care process. - Patients don’t want to use an activity tracker - Other: | 3,4,8-11 |
| 62 | I am open to start using activity trackers in COPD patients. | 7-point Likert scale (Strongly disagree- Strongly agree) | 1,2,8-10 |
| 63 | I am planning to start using activity trackers in COPD patients. | 7-point Likert scale (Strongly disagree- Strongly agree) | 3,4 |
| 64 | I think activity trackers should be used in COPD patients. | 7-point Likert scale (Strongly disagree- Strongly agree) | 3,4 |
| 65 | I have sufficient knowledge to (start to) use activity trackers. | 7-point Likert scale (Strongly disagree- Strongly agree) | 1,2,8-10 |
| 66 | I have sufficient knowledge to recommend commercially available activity trackers to my patients regarding price, functions and quality. | 7-point Likert scale (Strongly disagree- Strongly agree) | 5,8-10 |
| 67 | I trust I can use activity trackers in COPD patients. | 7-point Likert scale (Strongly disagree- Strongly agree) | 3,4,8-10 |
| 68 | I am capable to draft personalized physical activity goals together with my patient. | 7-point Likert scale (Strongly disagree- Strongly agree) | 5 |
| 69 | I am capable to teach my patients about the functions, use and interpretations of activity trackers. | 7-point Likert scale (Strongly disagree- Strongly agree) | 5,10 |
| 70 | My colleagues are open to (start) using an activity tracker. | 7-point Likert scale (Strongly disagree- Strongly agree) | 1,2,10 |
| 71 | My colleagues have sufficient skills and knowledge to (start) using activity trackers. | 7-point Likert scale (Strongly disagree- Strongly agree) | 1,2,10 |
| 72 | My COPD patients are open to (start) using activity trackers. | 7-point Likert scale (Strongly disagree- Strongly agree) | 1,2,10 |
| 73 | My COPD patients think it is important to (start) using activity trackers. | 7-point Likert scale (Strongly disagree- Strongly agree) | 5,10 |
| 74 | My COPD patients have sufficient skills and knowledge to (start) using an activity tracker. | 7-point Likert scale (Strongly disagree- Strongly agree) | 1,2,10 |
| 75 | My COPD patients experience commercially available activity trackers as technical complex. | 7-point Likert scale (Strongly disagree- Strongly agree) | 5,10 |
| 76 | My COPD patients are more aware of their functioning by using an activity tracker. | 7-point Likert scale (Strongly disagree- Strongly agree) | 3,4 |
| 77 | The use of activity trackers is part of our mission and vision and is incorporated into our policy. | 7-point Likert scale (Strongly disagree- Strongly agree) | 1,2,8,9 |
| 78 | The use of activity trackers fits in our care process. | 7-point Likert scale (Strongly disagree- Strongly agree) | 1,2,8,9 |
| 79 | There is sufficient support and involvement from the management when using activity trackers. | 7-point Likert scale (Strongly disagree- Strongly agree) | 1,2,8,9 |
| 80 | In our centre, there is someone who guides the implementation process of activity trackers. | 7-point Likert scale (Strongly disagree- Strongly agree) | 1,2,9 |
| 81 | In our centre, there is sufficient education/coaching for the healthcare professionals about activity trackers. | 7-point Likert scale (Strongly disagree- Strongly agree) | 1,2,8,9 |
| 82 | In our centre, there is sufficient time to try to use activity trackers. | 7-point Likert scale (Strongly disagree- Strongly agree) | 1,2,8,9 |
| 83 | In our centre, the use of activity trackers is incorporated in our electronic patients' files. | 7-point Likert scale (Strongly disagree- Strongly agree) | 1,2,8,9 |
| 84 | Activity trackers are provided to our patients. | - No - Yes; for how many days? | 5,8,9,11 |
| 85 | In our centre, there are sufficient activity trackers. | - No - Yes | 5,8,9,11 |
| 86 | To my centre, comparable healthcare centres are using activity trackers. | 7-point Likert scale (Strongly disagree- Strongly agree) | 1,2,9 |
| 87 | External organizations are obligating the use of activity trackers. | 7-point Likert scale (Strongly disagree- Strongly agree) | 1,2,9 |
| 88 | In our centre, the implementation of activity trackers was prepared and planned. | 7-point Likert scale (Strongly disagree- Strongly agree) | 1,2,9 |
| 89 | In our centre, we started using activity trackers on a trial basis and started to adapted our care process. | 7-point Likert scale (Strongly disagree- Strongly agree) | 1,2,9 |
| 99 | In our centre relevant persons were involved in the implementation process: healthcare professionals, patients (representatives), supporting professionals (policy). | 7-point Likert scale (Strongly disagree- Strongly agree) | 1,2,9 |
| 100 | In our centre, the use of activity tracker are frequently evaluated and improved. | 7-point Likert scale (Strongly disagree- Strongly agree) | 1,2,9 |
| 101 | The following factors stimulate the use of activity trackers during the clinical reasoning in patients with COPD: | Multiple-choice:   - Costs - Time investment for the therapist - Reliability and validity - User-friendliness - Motivation for the patient - The added value for the clinical reasoning - Other: | 5,8,-11 |
| 102 | The following factors obstruct the use of activity trackers during the clinical reasoning in patients with COPD: | - I have no knowledge about the use of activity trackers - Bad experiences with activity trackers - No added value to the care process. - Patients don’t want to use an activity tracker - Other: | 5,8-11 |

1. Beurskens A, Swinkels R, Wittink H, van Peppen R, Vermeulen H. Meten in de praktijk. Houten; 2020.

2. Damschroder LJ, Aron DC, Keith RE, Kirsh SR, Alexander JA, Lowery JC. Fostering implementation of health services research findings into practice: a consolidated framework for advancing implementation science. *Implement Sci* 2009; **4**: 50.

3. Stevens JGA. Ready for Goal setting? Maastricht: Maastricht University; 2017.

4. Ajzen I. The theory of planned behavior. *Organizational Behavior and Human Decision Processes* 1991; **50**(2): 179-211.

5. Ummels D, Beekman E, Moser A, Braun SM, Beurskens AJ. Patients’ experiences with commercially available activity trackers embedded in physiotherapy treatment: a qualitative study. *Disability and Rehabilitation* 2019: 1-9.

6. Vreeken HL, Beekman E, Van Doormaal M, Post MHL, Meerhoff GA, Spruit MA. KNGF-Richtlijn COPD. Amersfoort: Royal Dutch Society for Physiotherapy (KNGF), 2020.

7. Spruit MA et al. Profiling of Patients with COPD for Adequate Referral to Exercise-Based Care: The Dutch Model. *Sports Med*. 2020 Aug;50(8):1421-1429

8. Schreiweis B, Pobiruchin M, Strotbaum V, Suleder J, Wiesner M, Bergh B. Barriers and Facilitators to the Implementation of eHealth Services: Systematic Literature Analysis. *J Med Internet Res*. 2019 Nov 22;21(11):e14197.

9. Ross J, Stevenson F, Lau R, Murray E. Factors that influence the implementation of e-health: a systematic review of systematic reviews (an update). *Implement Sci*. 2016 Oct 26;11(1):146.

10. Kruse CS, Karem P, Shifflett K, Vegi L, Ravi K, Brooks M. Evaluating barriers to adopting telemedicine worldwide: A systematic review. *J Telemed Telecare*. 2018 Jan;24(1):4-12.

11. Granja C, Janssen W, Johansen MA. Factors Determining the Success and Failure of eHealth Interventions: Systematic Review of the Literature. *J Med Internet Res*. 2018 May 1;20(5):e10235.
